# Recent Highlights of Research on Androgen Receptors in Women

**DOI:** 10.34763/devperiodmed.20172101.0712

**Published:** 2017-05-29

**Authors:** Kamil Zaręba, Iwona Sidorkiewicz

**Affiliations:** 1 Department of Gynecology and Gynecologic Oncology, Medical University of Białystok, Białystok, Poland; 2 Department of Reproduction and Gynecological Endocrinology, Medical University of Białystok, Białystok, Poland

**Keywords:** androgen receptors, androgens, hyperandrogenism, protein splice variants, women

## Abstract

In this brief review we present an outline of the current state of research on examples of hyperandrogenism that can be strongly associated with diverse modifications in the androgen signaling pathway. We discuss the most prominent clinical features of androgen excess and correlate them with studies on androgen receptor (AR) alterations. For the first time we summarize the confirmed localizations of all known AR receptors in women. The knowledge of ARs may be the basis for AR-targeted therapies of androgenic disorders in women, including malignancy, as it has recently been demonstrated for triple-negative breast cancer. Moreover, we summarize the structure and characterization of key AR splice variants, which could be involved in the androgenization in women.

## Introduction

Hyperandrogenism in women is a highly important clinical and social problem affecting 5-10% of the population of reproductive age [1, 2]. Often described as androgen excess, this disorder encompasses a wide array of manifestations such as: polycystic ovary syndrome (PCOS), idiopathic hirsutism, hirsutism and hyperandrogenemia, non-classical congenital adrenal hyperplasia, hyperandrogenism, insulin resistance, acanthosis nigricans (HAIR-AN), ovarian or adrenal androgen-secreting neoplasms, androgenic drug intake, Cushing’s syndrome, and hyperprolactinemia [3]. Importantly, the above list may not be complete. Moreover, it includes disorders with normal and modified androgen production and metabolism, as well as those with altered androgen receptor (AR) or its defective signaling pathways. Overall, hyperandrogenism includes both clinical and biochemical androgen excess. In this paper we focus on the crucial role of ARs in the pathophysiological and clinical manifestations of hyperandrogenism in women.

In recent years the pace of research on ARs has been gaining a particular momentum since more and more scientific evidence supports their importance for tissue homeostasis in women. For example, roles of ARs have been studied in such complex processes as reproductive failure (on ovarian, oocytic, and uterine levels), endometrial hyperplasia, and cancer [4, 5, 6, 7]. However, few studies analyzed specific truncated forms of the AR, called splice variants.

## Full-length androgen receptor versus its splice variants

The expression of full-length AR (AR-FL) has been proven across most of the female reproductive tissues, which emphasizes its apparent functional importance, especially within the endometrium [8]. Tuckerman et al. established that androgens exert a direct effect on endometrial function by demonstrating the presence of ARs in cultured endometrial cells, and showing that this effect is nullified after AR inhibitor treatment [9]. The human AR is a transcription regulator belonging to the nuclear receptor family. AR gene structure, mature spliced mRNA, and protein domains bear a resemblance to other family members: estrogen receptors and progesterone receptors. *AR* includes 8 different exons forming the AR protein consisting of 4 domains, each of specialized function: 1) the N-terminal domain (NTD); 2) the DNA binding domain (DBD); 3) a hinge region (HR); and 4) the C-terminal ligand binding domain (LBD) [10]. When AR is present in the cytosol, it remains in its transcriptionally inactive form stabilized by molecular chaperone heat shock protein 90 (HSP90). Once a ligand (such as testosterone or dihydrotestosterone) attaches to LBD, AR dissociates from HSP90. Subsequently, it dimerizes and translocates into the nucleus, where it binds to the androgen response element in the regulatory region of its target genes, thus modulating their expression [11].

**Fig. 1:**
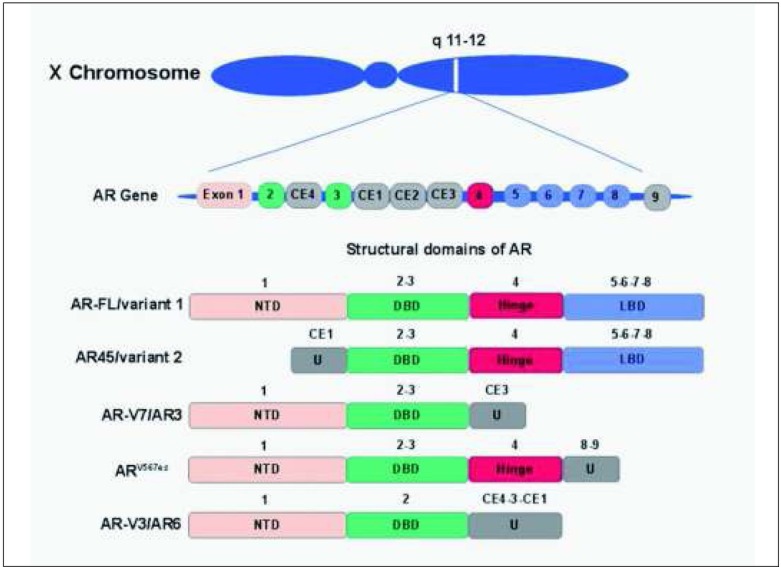
The *AR* gene and its key protein transcripts. The *AR* is located on the X chromosome (*locus*: Xq11-Xq12) and includes 8 exons encoding 4 distinct domains: N-terminal domain (NTD), DNA-binding domain (DBD), hinge region, and ligand-binding domain (LBD). The above simplified scheme distinguishes: exons 1 to 8 of full-length AR (AR-FL), cryptic exons (i.e., CE1-4) of AR splice variants (AR-Vs) and AR45, and exon 9 of Ar^V567es^. U - unique sequences not found in AR-FL. Adapted from [12].

Figure 1 presents the current understanding of the *AR* structure and the most prevalent AR transcripts compositions [12].

It should be noted that AR-FL coexists in tissues with its truncated isoforms lacking LBD that are referred to as AR splice variants (AR-Vs), and with variant 2 of the AR named AR45. The truncated variants are products of alternative splicing of the human *AR* gene [13, 14]. As of now, 17 different AR splice variants have been characterized and labeled [15]. Overall, AR-Vs fall into two categories depending on their structure: the truncated variants, and the exon skipping variants [16]. It has been established that AR-Vs differ significantly in function both from AR-FL and among themselves depending on alterations in their structure. First, the most frequent alteration is the loss of LBD, which results in ligand-independent constitutive actions [17]. Secondly, the actions of AR-Vs can be mediated through heterodimerization with the wild-type receptor, as well as homodimerization, therefore a potential interplay between the two types has been discussed in depth [18, 19]. Finally, AR-V7 (also known as AR3) and AR^V567es^ (also known as AR-V12) have been commonly identified in cancer tissue and ascribed an important role in cancer pathogenesis [15]. For example, strong correlations between AR-V7 and prostate cancer staging and prognosis have been established [20, 21]. Moreover, in prostate cancer cells, AR-V7-targeted specific protein immunoreactivity has been found to be greater than that of AR-FL. This observation was somewhat unexpected, because the expression of *AR-V7* gene in these cells is relatively less pronounced than that of the *AR-FL* gene. However, such a high yield protein transcription can be explained by excessive constitutive activity of AR-Vs, especially in the absence of any ligand, compared to AR-FL ligand-activated transcription [17, 22]. Both AR-V7 and AR-FL share impact on expression of at least 71 genes [23]. Nonetheless, recent research in mice highlighted a paramount functional difference: AR-V7 regulates its own unique set of genes that boost cell proliferation and division, whereas unique genes regulated by AR-FL are responsible mainly for cell differentiation and maturation [23].

Table I presents the known localization and characterization of ARs, including AR-Vs, in tissues of women [15, 24, 25, 26, 27].

**Table I:** The localization of ARs found in various tissues in women, and characterization of known AR-Vs [15,24-27]. NTD - N-terminal domain; DBD - DNA-binding domain; HR - hinge region; LBD - ligand-binding domain; CE - cryptic exon; ZF - zinc finger; U - unique N-terminal extension.

Name	Aliases	Splice junction in AR-Vs and other structural changes in AR45	Domains excluded	Confirmed tissue localization in women	Activity
AR-FL	AR wild-type	Reference structure	None	Ovary, uterus, endometrium, cervix, placenta, vagina, vulva, breast, cervix, placenta, vagina, skin	Ligand-stimulated
AR45	AR transcript variant 2	NTD replaced by U which is linked to the DBD, HR, and LBD of the AR	NTD	ovary, cervix, breast, placenta	Conditional
AR-V1	AR4	3/CE	HR to LBD	ovary, cervix, breast, placenta	Conditional
AR-V2	AR1/2/2B	3/3/CE1	HR to LBD		Constitutive
AR-V3	-	2/CE4	ZF2 to LBD	ovary, cervix, breast, placenta	Constitutive
AR-V4	AR5, AR1/2/3/2B	3/CE4	HR to LBD		Constitutive
AR-V5	-	3/CE2	HR to LBD		Constitutive
AR-V6	-	3/CE2	HR to LBD		Constitutive
AR-V7	AR3	3/CE3	HR to LBD	ovary, cervix, breast, placenta	Constitutive
AR-V8	-	3/intron 3	HR to LBD		Constitutive
AR-V9	-	3/CE5	HR to LBD	ovary, cervix, breast, placenta	Conditional
AR-V10	-	3/intron 3	HR to LBD		Constitutive
AR-V11	-	3/intron 3	HR to LBD		Unknown
AR-V12	ARV567es	4/8/9	LBD (disrupted)		Constitutive
AR-V13	-	6/9	LBD (disrupted)		Inactive
AR-V14	-	7/9			Unknown
AR-V15	-	6/9	LBD (disrupted)		Unknown
AR-V16	-	8/9			Unknown
AR-V17	-	8/9			Unknown
AR-V18	-	6/9			Unknown

## Androgen receptors and clinical features of hyperandrogenism

In the light of ARs abundance in women (Tab. I), their pivotal role in the pathogenesis of hyperandrogenism should be thoroughly analyzed. Hence, it is important to distinguish the most common clinical manifestations facilitating the diagnosis of this disorder and select appropriate patients for further in-depth hormonal and receptor validation. One of such manifestations is hirsutism which affects up to 15% of all women and 70-80% of women with hyperandrogenism [1, 2]. Visual assessment methods of hair type growth, such as Ferriman-Gallwey score [28], are efficient in determining the severity of hirsutism and differentiating it from hypertrichosis (excess growth of androgen independent hair) [29]. Virilization represents a rapid process in which severe clinical features of marked androgen excess occur. As a part of a broader spectrum it can include: hirsutism, acne, androgenic alopecia, enlargement of the clitoris, deepening of the voice, increased muscle mass, decreased breast size, and frequently amenorrhea [3]. Furthermore, hyperandrogenism correlates with numerous metabolic alterations, for example: hyperinsulinemia, insulin resistance, hyperglycemia, dyslipidemia, and increased risk of atherosclerosis [30, 31, 32]. These features represent the core components of metabolic syndrome. Importantly, changes of AR in hyperandrogenism must be mediated via ARs-controlled pathway(s). For example, Apparao et al. established that women with PCOS exhibited elevated endometrial AR expression compared to controls. Further, AR in endometrial cell lines was upregulated not only by androgens but also by estrogens [32]. Another example of AR significance is the confirmed increased risk for breast cancer in premenopausal women with elevated serum concentrations of testosterone, androstenedione and dehydroepiandrosterone sulfate [33]. Therefore, novel breast cancer therapies are targeting the AR for its observed expression in the three main breast cancer subtypes [34, 35, 36]. Importantly, AR has already been demonstrated as a promising target for antiandrogen therapies in the most aggressive triple-negative breast cancer [37].

## Other correlates of androgen receptor involvement

To date, AR-Vs have been studied to some extent in human breast cancer cell lines and ovarian granulosa cells (GCs) [27, 38]. Recent research by Wang et al. identified two alternative splice variants (exon 3 deletion isoform, and 69 bp insertion into intron 2 isoform) in GCs from PCOS women. Moreover, there was no coexpression of both these isoforms in any of the patient studied. This investigation also described up-regulation of total AR mRNA in GCs of PCOS patients compared with controls; no expression of AR-Vs was observed in the control group. Since the levels of AR-FL were found reduced, the authors ascribed this up-regulation primarily to the increased mRNA levels of AR-Vs [38]. In this study AR-Vs were strongly associated with marked hyperandrogenism and abnormalities in folliculogenesis which, on the other hand, were absent in all control subjects. Therefore, Wang et al. claim that alternative splicing of the AR in GCs could be a major causative mechanism in PCOS (38). In contrast, in the opinion of Walters et al., increased AR-Vs expression in PCOS is considered rather the effect of the aberrant hyperandrogenic follicular milieu, rather than its cause [39].

All in all, we are currently experiencing an exciting era of new discoveries in the field of ARs and their possible multiple roles in women. Our brief review highlights that the knowledge of AR signaling and receptor structure alterations is crucial for the understanding of the pathogenesis of many androgenic disorders. Therefore, future scientific efforts targeted to further decipher the AR significance in women’s health and disease will be of utmost importance.

## References

[1] Azziz R, Sanchez LA, Knochenhauer ES, Moran C, Lazenby J, Stephens KC, Taylor K, Boots LR (2004). Androgen excess in women: experience with over 1000 consecutive patients. J Clin Endocrinol Metab.

[2] Carmina E, Rosato F, Jannì A, Rizzo M, Longo RA. (2006). Extensive clinical experience: relative prevalence of different androgen excess disorders in 950 women referred because of clinical hyperandrogenism. J Clin Endocrinol Metab.

[3] Yildiz BO (2006). Diagnosis of hyperandrogenism: clinical criteria. Best Pract Res Clin Endocrinol Metab.

[4] Gibson DA, Simitsidellis I, Cousins FL, Critchley HOD, Saunders PTK (2016). Intracrine androgens enhance decidualization and modulate expression of human endometrial receptivity genes. Sci Rep.

[5] Gibson DA, Simitsidellis I, Collins F, Saunders PTK (2014). Evidence of androgen action in endometrial and ovarian cancers. Endocr Relat Cancer.

[6] Cochrane DR, Bernales S, Jacobsen BM, Cittelly DM, Howe EN, D’Amato NC, Spoelstra NS, Edgerton SM, Jean A, Guerrero J, Gómez F, Medicherla S, Alfaro IE, McCullagh E, Jedlicka P, Torkko KC, Thor AD, Elias AD, Protter AA, Richer JK (2014). Role of the androgen receptor in breast cancer and preclinical analysis of enzalutamide. Breast Cancer Res.

[7] Mylonas I, Makovitzky J, Friese K, Jeschke U (2009). Immunohistochemical labelling of steroid receptors in normal and malignant human endometrium. Acta Histochem.

[8] Takeda H, Chodak G, Mutchnik S, Nakamoto T, Chang C (1990). Immunohistochemical localization of androgen receptors with mono- and polyclonal antibodies to androgen receptor. J Endocrinol.

[9] Tuckerman EM, Okon MA, Li T-C, Laird SM (2000). Do androgens have a direct effect on endometrial function? An in vitro study. Fertil Steril.

[10] Gelmann EP (2002). Molecular biology of the androgen receptor. J Clin Oncol.

[11] Feldman BJ, Feldman D (2001). The development of androgen-independent prostate cancer. Nat Rev Cancer.

[12] Graham L, Schweizer MT (2016). Targeting persistent androgen receptor signaling in castration-resistant prostate cancer. Med Oncol.

[13] Guo Z, Yang X, Sun F, Jiang R, Linn DE, Chen H, Chen H, Kong X, Melamed J, Tepper CG, Kung H-J, Brodie AMH, Edwards J, Qiu Y (2009). A novel androgen receptor splice variant is up-regulated during prostate cancer progression and promotes androgen depletion-resistant growth. Cancer Res.

[14] Nyquist MD, Dehm SM (2013). Interplay between genomic alterations and androgen receptor signaling during prostate cancer development and progression. Horm Cancer.

[15] Azoitei A, Merseburger AS, Godau B, Hoda MR, Schmid E, Cronauer MV (2017). C-terminally truncated constitutively active androgen receptor variants and their biologic and clinical significance in castration-resistant prostate cancer. J Steroid Biochem Mol Biol.

[16] Sprenger CCT, Plymate SR (2014). The link between androgen receptor splice variants and castration-resistant prostate cancer. Horm Cancer.

[17] Xu J, Qiu Y (2016). Role of androgen receptor splice variants in prostate cancer metastasis. Asian J Urol.

[18] Xu D, Zhan Y, Qi Y, Cao B, Bai S, Xu W, Gambhir SS, Lee P, Sartor O, Flemington EK, Zhang H, Hu C-D, Dong Y (2015). Androgen receptor splice variants dimerize to transactivate target genes. Cancer Res.

[19] McCrea E, Sissung TM, Price DK, Chau CH, Figg WD (2016). Androgen receptor variation affects prostate cancer progression and drug resistance. Pharmacol Res.

[20] Welti J, Rodrigues DN, Sharp A, Sun S, Lorente D, Riisnaes R, Figueiredo I, Zafeiriou Z, Rescigno P, de Bono JS, Plymate SR. (2016). Analytical validation and clinical qualification of a new immunohistochemical assay for androgen receptor splice variant-7 protein expression in metastatic castration-resistant prostate cancer. Eur Urol.

[21] Ciccarese C, Santoni M, Brunelli M, Buti S, Modena A, Nabissi M, Artibani W, Martignoni G, Montironi R, Tortora G, Massari F (2016). AR-V7 and prostate cancer: The watershed for treatment selection?. Cancer Treat Rev.

[22] Hörnberg E, Ylitalo EB, Crnalic S, Antti H, Stattin P, Widmark A, Bergh A, Wikström P. (2011). Expression of androgen receptor splice variants in prostate cancer bone metastases is associated with castration-resistance and short survival. PLoS ONE.

[23] Guo H, Li Y, Luo M, Lin S, Chen J, Ma Q, Gu Y, Jiang Z, Gui Y (2015). Androgen receptor binding to an androgen-responsive element in the promoter of the Srsf4 gene inhibits its expression in mouse Sertoli cells. Mol Reprod Dev.

[24] Kimura N, Mizokami A, Oonuma T, Sasano H, Nagura H (1993). Immunocytochemical localization of androgen receptor with polyclonal antibody in paraffin-embedded human tissues. J Histochem Cytochem.

[25] Hodgins MB, Spike RC, Mackie RM, MacLean AB (1998). An immunohistochemical study of androgen, oestrogen and progesterone receptors in the vulva and vagina. Br J Obstet Gynaecol.

[26] Kohlberger PD, Joura EA, Bancher D, Gitsch G, Breitenecker G, Kieback DG (1998). Evidence of androgen receptor expression in lichen sclerosus: an immunohistochemical study. J Soc Gynecol Investig.

[27] Hu DG, Hickey TE, Irvine C, Wijayakumara DD, Lu L, Tilley WD, Selth LA, Mackenzie PI (2014). Identification of androgen receptor splice variant transcripts in breast cancer cell lines and human tissues. Horm Cancer.

[28] Ferriman D, Gallwey JD (1961). Clinical assessment of body hair growth in women. J Clin Endocrinol Metab.

[29] Rachoń D. (2012). Differential diagnosis of hyperandrogenism in women with polycystic ovary syndrome. Exp Clin Endocrinol Diabetes.

[30] Stanczyk FZ (2006). Diagnosis of hyperandrogenism: Biochemical criteria. Best Pract Res Clin Endocrinol Metab.

[31] Azziz R, Carmina E, Dewailly D, Diamanti-Kandarakis E, Escobar-Morreale HF, Futterweit W, Janssen OE, Legro RS, Norman RJ, Taylor AE, Witchel SF (2009). The Androgen Excess and PCOS Society criteria for the polycystic ovary syndrome: the complete task force report. Fertil Steril.

[32] Apparao KB, Lovely LP, Gui Y, Lininger RA, Lessey BA (2002). Elevated endometrial androgen receptor expression in women with polycystic ovarian syndrome. Biol Reprod.

[33] Kaaks R, Berrino F, Key T, Rinaldi S, Dossus L, Biessy C, Secreto G, Amiano P, Bingham S, Boeing H, Mesquita D, Bueno HB, Chang-Claude J, Clavel-Chapelon F, Fournier A, Gils V, Gonzalez CA, Gurrea AB, Critselis E, Khaw KT, Krogh V, Lahmann PH, Nagel G, Olsen A, Onland-Moret NC, Overvad K, Palli D, Panico S, Peeters P, Quirós JR, Roddam A, Thiebaut A, Tjønneland A, Chirlaque MD, Trichopoulou A, Trichopoulos D, Tumino R, Vineis P, Norat T, Ferrari P, Slimani N, Riboli E. (2005). Serum sex steroids in premenopausal women and breast cancer risk within the European prospective investigation into cancer and nutrition (EPIC). J Natl Cancer Inst.

[34] Chia K, O’Brien M, Brown M, Lim E (2015). Targeting the androgen receptor in breast cancer. Curr Oncol Rep.

[35] Iacopetta D, Rechoum Y, Fuqua SA (2012). The role of androgen receptor in breast cancer. Drug Discov Today Dis Mech.

[36] Mishra AK, Agrawal U, Negi S, Bansal A, Mohil R, Chintamani C, Bhatnagar A, Bhatnagar D, Saxena S (2012). Expression of androgen receptor in breast cancer & its correlation with other steroid receptors & growth factors. Indian J Med Res.

[37] Rampurwala M, Wisinski KB, O’Regan R. (2016). Role of the androgen receptor in triple-negative breast cancer. Clin Adv Hematol Oncol.

[38] Wang F, Pan J, Liu Y, Meng Q, Lv P, Qu F, Ding G-L, Klausen C, Leung PCK, Chan HC, Yao W, Zhou C-Y, Shi B, Zhang J, Sheng J, Huang H (2015). Alternative splicing of the androgen receptor in polycystic ovary syndrome. Proc Natl Acad Sci US A.

[39] Walters KA, Handelsman DJ (2016). Androgen receptor splice variants and polycystic ovary syndrome: cause or effect?. Asian J Androl.

